# Studies of Protein Binding to Biomimetic Membranes Using a Group of Uniform Materials Based on Organic Salts Derived From 8-Anilino-1-naphthalenesulfonic Acid

**DOI:** 10.1177/00037028241249768

**Published:** 2024-05-15

**Authors:** Ana M.O. Azevedo, Cláudia Nunes, Tânia Moniz, Rocío L. Pérez, Caitlan E. Ayala, Maria Rangel, Salette Reis, João L.M. Santos, Isiah M. Warner, M. Lúcia M.F.S. Saraiva

**Affiliations:** 1 LAQV, REQUIMTE, Departamento de Ciências Químicas, 131671 Faculdade de Farmácia, Universidade do Porto, Porto, Portugal; 2 LAQV, REQUIMTE, Departamento de Química e Bioquímica, 131671 Faculdade de Ciências, Universidade do Porto, Porto, Portugal; 3 LAQV, REQUIMTE, Instituto de Ciências Biomédicas Abel Salazar, 131671 Universidade do Porto, Porto, Portugal; 4 Department of Chemistry, 5779 Louisiana State University, Baton Rouge, Louisiana, USA; 5 Department of Chemistry and Biochemistry, 7604 Georgia Southern University, Statesboro, Georgia, USA

**Keywords:** 8-anilino-1-naphthalenesulfonic acid, ANS‌, group of uniform materials based on organic salts, ‌GUMBOS, liposome, protein, protein-membrane binding

## Abstract

Tuning the 8-anilino-1-naphthalenesulfonic acid (ANS) structure usually requires harsh conditions and long reaction times, which can result in low yields. Herein, ANS was modified to form an ANS group of uniform materials based on organic salts (GUMBOS), prepared with simple metathesis reactions and distinct cations, namely tetrabutylammonium (N_4444_), tetrahexylammonium (N_6666_), and tetrabutylphosphonium (P_4444_). These ANS-based GUMBOS were investigated as fluorescent probes for membrane binding studies with four proteins having distinct physicochemical properties. Liposomes of 1,2-dimyristoyl-*sn*-glycero-3-phosphocholine were employed as membrane models as a result of their ability to mimic the structure and chemical composition of cell membranes. Changes in fluorescence intensity were used to monitor protein binding to liposomes, and adsorption data were fitted to a Freundlich-like isotherm. It was determined that [N_4444_][ANS] and [P_4444_][ANS] GUMBOS have enhanced optical properties and lipophilicity as compared to parent ANS. As a result, these two GUMBOS were selected for subsequent protein-membrane binding studies. Both [N_4444_][ANS] and [P_4444_][ANS] GUMBOS and parent ANS independently reached membrane saturation within the same concentration range. Furthermore, distinct fluorescence responses were observed upon the addition of proteins to each probe, which demonstrates the impact of properties such as lipophilicity on the binding process. The relative maintenance of binding cooperativity and maximum fluorescence intensity suggests that proteins compete with ANS-based probes for the same membrane binding sites. Finally, this GUMBOS-based approach is simple, rapid, and involves relatively small amounts of reagents, making it attractive for high-throughput purposes. These results presented herein can also provide relevant information for designing GUMBOS with ameliorated properties.

## Introduction

The binding of proteins to biological membranes plays an important role in a multitude of cellular processes, including signal transduction, lipid metabolism, and vesicle trafficking.^1,2^ Additionally, protein–lipid interactions are understood to be involved in pathological conditions ranging from inflammation to cancer.^3,4^ For example, it has been proposed that oligomeric beta-amyloid binds to GM1 ganglioside and decreases membrane fluidity, thereby stimulating proteolytic cleavage of the amyloid precursor protein. This effect can result in beta-amyloid accumulation and the formation of neuritic plaques, which are characteristic hallmarks of Alzheimer's disease.^5,6^

Various membrane model systems have been employed to study interactions between proteins and lipids, including liposomes, lipid monolayers, and supported lipid bilayers.^7,8^ Liposomes are among the most commonly used models as a result of their ability to closely resemble the structure and properties of cell membranes. In fact, these model systems are composed of a phospholipid bilayer enclosing an aqueous compartment that can mimic the anisotropic environment of biological membranes.^9–11^ Phosphatidylcholine accounts for 39% of plasma membrane phospholipids and is predominant in eukaryotic cells. As such, 1,2-dimyristoyl-*sn*-glycero-3-phosphocholine (DMPC) was chosen for the preparation of liposomes in the present study.^12,13^

The anionic dye, 8-anilino-1-naphthalenesulfonic acid (ANS), has been widely employed as a fluorescent probe for protein analyses. Several research groups have demonstrated the ability of this probe to determine the surface hydrophobicity of proteins, detect conformational changes, and characterize protein binding sites.^14–16^ ANS has also been extensively used as a reporter for studying interactions of small molecules (e.g., drugs) with the liposomes’ surface.^17–19^ However, to the best of our knowledge, there are only a few reports on the use of ANS dye to study protein-membrane binding and this is the first that combines ANS with liposomes to probe different proteins.^20,21^ It is also worth noting that this probe is mostly located at the membrane surface since its naphthalene moiety only partially enters the hydrophobic core.^22,23^ Tuning the ANS structure can include extensive resource investment, harsh conditions, and long reaction times, which can result in low yields.^24,25^ In contrast, we hypothesized that the group of uniform materials based on organic salts (GUMBOS) approach would allow easy and cost-effective development of ANS derivatives with improved and targeted properties. GUMBOS are solid-phase organic salts that are typically composed of bulky and asymmetric ions and can be prepared using simple metathesis reactions, without the need for extensive resource input and time-consuming purification steps.^26–28^ Moreover, GUMBOS properties including, but not limited to, lipophilicity can be readily tuned by changing the cation–anion combination, thereby avoiding complicated chemical modifications.^29,30^

In this study, ANS was converted into GUMBOS through single-step metathesis reactions using cations with different central atoms and alkyl side chains, namely tetrabutylammonium (N_4444_), tetrahexylammonium (N_6666_), and tetrabutylphosphonium (P_4444_). The properties of ANS-based GUMBOS were examined and their potential applications as fluorescent probes for membrane binding studies were investigated. For this purpose and due to differences in surface hydrophobicity, lysozyme (Lyz), myoglobin (Mb), α-chymotrypsin (α-ChT), and ribonuclease A (RNAse A) were selected as model proteins. Changes in fluorescence intensities were used to study protein binding to DMPC liposomes. Experiments were also conducted with the parent ANS dye to further confirm the potential of this GUMBOS approach.

## Material and Methods

### Reagents and Solvents

Tetrabutylammonium bromide ([N_4444_][Br]), tetrahexylammonium bromide ([N_6666_][Br]), Lyz from chicken egg white, Mb from equine heart, α-ChT from bovine pancreas, RNAse A from bovine pancreas, N-(2-hydroxyethyl)piperazine-N′-(2-ethanesulfonic acid) (HEPES) hemisodium salt, hexadecylphosphocholine (HePC), and dichloromethane (DCM) were all purchased from Sigma-Aldrich. Tetrabutylphosphonium bromide ([P_4444_][Br]) was acquired from Fluka, and DMPC was obtained from Avanti Polar Lipids. ANS ammonium salt ([NH_4_][ANS]) was purchased from Tokyo Chemical Industry (TCI America) and ANS from Invitrogen. Sodium chloride (NaCl) was obtained from PanReac AppliChem. Chloroform and methanol were purchased from VWR International, and acetonitrile (ACN) from Riedel-de Haën. Deuterated methanol (CD_3_OH, 99.8%) with 0.03% (v/v) tetramethylsilane was acquired from Eurisotop.

The HEPES buffer, 0.01 mol L^–1^ (pH 7.4) was obtained by dissolving HEPES and NaCl in high-purity water (Milli-Q, 18.2 MΩ·cm), and used for the preparation of HePC stock suspension. This suspension (600 μmol L^–1^) was further diluted in HEPES buffer to a concentration of 100 μmol L^–1^. Protein stock and working solutions (2 mg mL^–1^ and 300 μg mL^–1^, respectively) were prepared in the same buffer. ANS-based GUMBOS was dissolved in ACN, protected from light, and used immediately after preparation. Before membrane partition and protein–lipid binding studies, these stock solutions were further diluted in HEPES buffer to obtain a concentration of 450 μmol L^–1^ and 100 μmol L^–1^, respectively.

### Instrumentation

Nuclear magnetic resonance (NMR) spectra were recorded using a Bruker Avance III 400 operating at 400.15 MHz for proton (^1^H) and 100.62 MHz for carbon (^13^C). The spectrometer was equipped with a pulse gradient unit, capable of producing a z-gradient of 50.0 G cm^–1^. Two-dimensional (2D) ^1^H–^13^C heteronuclear single quantum coherence (HSQC) and ^1^H–^13^C heteronuclear multiple bond coherence (HMBC) spectra were acquired and processed using Bruker's TopSpin software v.4.0.1. NMR samples were prepared by dissolving synthesized GUMBOS in CD_3_OH. Coupling constants (*J*) and chemical shifts (δ) were reported in hertz (Hz) and parts per million (ppm), respectively. Peak multiplicities were abbreviated as follows: bs, broad singlet; dd, double doublet; t, triplet; q, quartet; quint, quintet; and m, multiplet. NMR analyses were conducted at Laboratório de Análise Estrutural, Centro de Materiais da Universidade do Porto, Portugal.

High-resolution mass spectrometry (HRMS) measurements were performed at the Louisiana State University Mass Spectrometry facility using an Agilent 6230 B-TOF liquid chromatography–mass spectrometry system, operated in both positive and negative ionization modes. A Bruker Tensor 27 spectrometer, equipped with a Pike MIRacle attenuated total reflection accessory, was used to obtain Fourier transform infrared spectroscopy (FT-IR) spectra in the range of 4000 to 400 cm^–1^. Resolution was set to 4 cm^–1^ and the number of scans to 32.

Ultraviolet–visible (UV–Vis) and fluorescence spectroscopic studies were performed using a Jasco V-660 spectrophotometer and a Jasco FP-6500 spectrofluorometer, both equipped with a 1 cm quartz cuvette. UV–Vis spectra were recorded in the range of 250–500 nm, and fluorescence emission spectra from 390 to 650 nm upon excitation at 370 nm.

The thermal stability of [N_4444_][ANS] and [P_4444_][ANS] was investigated using a TA Discovery TGA 550 instrument. GUMBOS were heated from 25 to 595 °C using a ramp rate of 10 °C min^–1^. The melting points of these compounds were also determined using a MEL-TEMP apparatus. Membrane partition and competitive binding assays were performed on a BioTek Cytation 3 imaging reader.

### Synthesis and Characterization of GUMBOS

**Figure 1.**
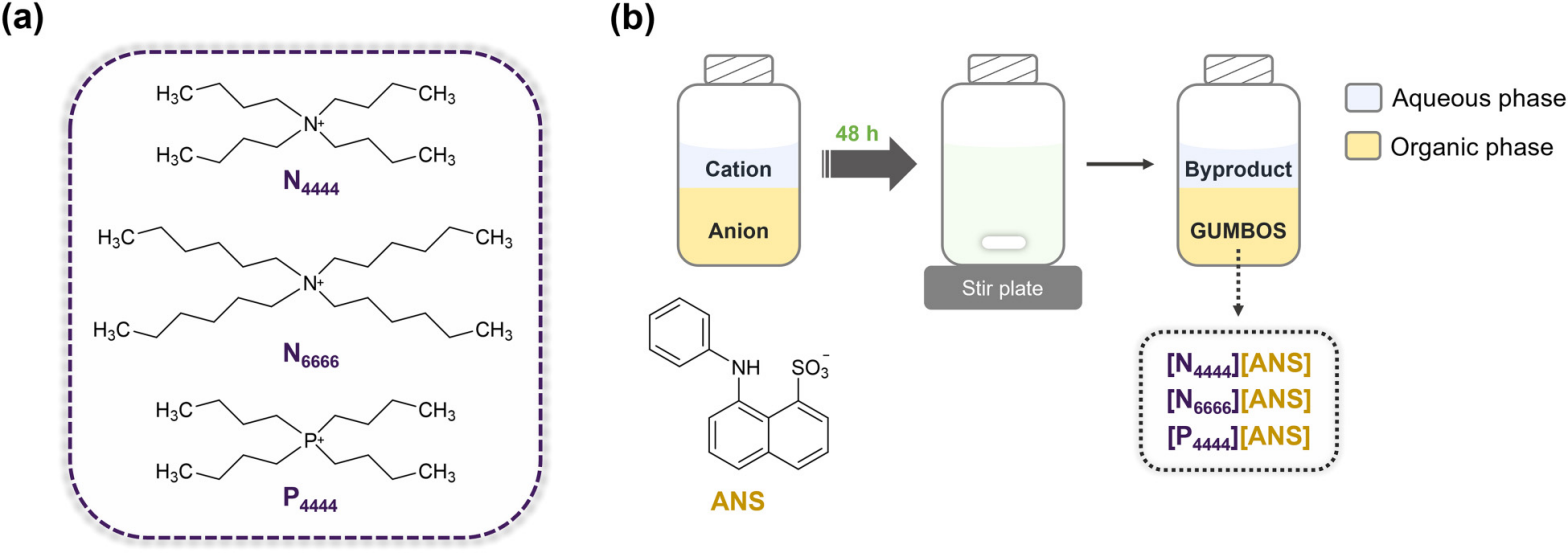
(a) Chemical structures of the cations used to form ANS-based GUMBOS. (b) Schematic representation of GUMBOS syntheses.

The ANS-based GUMBOS were synthesized using simple ion-exchange metathesis reactions, where the ammonium cation of parent ANS dye was replaced with N_4444_^+^, N_6666_^+^, and P_4444_^+^ (Figure 1a). Following a previously described procedure, 5 mL of water containing the desired cation was added to 10 mL of a DCM solution of [NH_4_][ANS] (1.1 : 1 molar ratio).^31,32^ The biphasic mixture was stirred for 48 h in the dark at room temperature, after which the top layer (aqueous phase) was discarded (Figure 1b). The DCM phase was then washed several times with water to minimize any remaining halogenated byproduct (i.e., NH_4_Br). Subsequently, DCM was evaporated, and residual water was removed by freeze-drying. The resulting GUMBOS were structurally characterized using NMR, HRMS, and FT-IR. Additionally, spectral properties were investigated using UV–Vis and fluorescence spectroscopies. Physical characteristics such as lipophilicity, thermal stability, and melting point, were also examined using derivative UV–Vis spectrophotometry, thermogravimetric analysis (TGA), and melting point determination, respectively.

### Group of Uniform Materials Based on Organic Salts (GUMBOS) Partitioning

The HePC micelle/buffer partition coefficients (*K*_p_) of synthesized GUMBOS and parent ANS dye were assessed using a derivative UV–Vis spectrophotometry protocol as previously described.^33,34^ Samples were prepared by adding 50 µL of each probe solution (final concentration = 75 µmol L^–1^) to increasing volumes of HePC suspension to obtain concentrations ranging from 0 to 200 µmol L^–1^. In parallel, references were obtained by replacing GUMBOS and ANS with HEPES buffer containing the same percentage of ACN as samples (<3%). Both samples and references were incubated at 37 °C for 30 min, after which absorption spectra were recorded in the spectral range of 300–600 nm. Experimental results were analyzed using the Excel spreadsheet (*K*_p_ calculator) developed by Magalhaes et al.,^33^ which first subtracts each reference spectrum from the corresponding sample spectrum, and then generates the spectrum of derivatives. Second derivative values, measured at the wavelength where light scattering was negligible (λ = 348 nm), were plotted against the concentrations of HePC. The value of *K*_p_ (mol L^–1^) was determined by fitting Eq. 1 to experimental data using a nonlinear least squares regression method:^33^

(1) $$D_{T}=D_{W}+\frac{\left(D_{L}-D_{W})K_{p}[L\right]}{1\;+K_{p}[L]}$$

where *D_T_*, *D_W_*, and *D_L_* represent the second derivative intensities obtained from absorbance values of the total amount of GUMBOS, and from GUMBOS in the aqueous and lipid phases, respectively. The parameter [*L*] is the HePC concentration and *K*_p_ is the partition coefficient (both expressed in mol L^−1^). The dimensionless value (log *D*) was calculated by dividing the molar partition coefficient by HePC molar volume (0.406 L mol^–1^).^33^

### Preparation of DMPC Liposomes

Liposomes were prepared using a thin-film hydration method in combination with extrusion.^35,36^ First, DMPC was dissolved into a mixture of chloroform and methanol (9 : 1, v/v), and then the organic solvent mixture was evaporated using a nitrogen stream and vacuum. The resulting lipid film was dispersed in HEPES buffer at 40 °C (i.e., above DMPC main phase transition temperature) and vortexed to form multilamellar vesicles (MLVs).^37,38^ Subsequently, the MLVs suspension was extruded 10 times through a polycarbonate filter with a pore size of 100 nm to obtain large unilamellar vesicles (LUVs).

### Protein-Membrane Binding Studies

The binding of proteins to liposomes was studied using fluorescence titration with ANS-based GUMBOS and the parent dye. Briefly, 20 μL of each protein sample (i.e., Lyz, Mb, α-ChT, and RNAse A) were mixed with 30 μL of DMPC LUVs and increasing volumes of ANS-based GUMBOS or parent ANS (final concentration = 0–80 µmol L^–1^). HEPES buffer was added to achieve a final volume of 300 μL. Protein and lipid concentrations were fixed at 20 μg mL^–1^ and 10 μmol L^–1^, respectively. References were prepared in the absence of GUMBOS or parent dye, which were replaced with HEPES buffer using the same percentage of organic solvent (ACN) as the corresponding samples. Following a 5 min incubation period at 37 °C, fluorescence emission spectra were recorded from 380 to 650 nm, using 350 nm excitation. Absorbance values were measured at the excitation wavelength to correct fluorescence intensities using Eq. 2, minimizing inner filter effects.^39^ This effect results from protein absorption at the fluorophore's excitation wavelength and decreases the effective intensity of the exciting light beam, which ultimately leads to a reduction in the overall fluorescence intensity. Thus,

(2) $$I_{\mathrm{corr}}=I\frac{A_{T}}{A_{F}}\frac{1-10^{-A_{F}}}{1-10^{-A_{T}}}$$

where *I*_corr_ is the corrected fluorescence intensity and *I* the experimental fluorescence. The parameters *A_T_* and *A_F_* represent the absorbance of the sample with and without protein, respectively.

Finally, the impact of proteins on the membrane binding of ANS-based GUMBOS was investigated by fitting corrected adsorption data to an equation derived from the Freundlich isotherm (Eq. 3):^40^

(3) $$[\mathrm{GUMBOS}{]}_{B}=C_{\max}\frac{{\left(K_{b}{\left[\mathrm{GUMBOS}\right]}_{\infty }\right)}^{b}}{1+{\left(K_{b}{\left[\mathrm{GUMBOS}\right]}_{\infty }\right)}^{b}}$$

where *K_b_* is the binding constant, *C*_max_ is the maximum fluorescence intensity, *b* is the cooperativity of the binding process, and *B* and ∞ represent the concentrations of the bound and free forms of GUMBOS. This same equation was applied to experimental data obtained using the parent ANS dye.

## Results and Discussion

### Structural Characterization of the Synthesized GUMBOS

The structures of ANS-based GUMBOS were determined using combined analysis of NMR (1D and 2D), HRMS, and FT-IR data. Integration of peaks in ^1^H NMR spectra confirmed the desired 1 : 1 stoichiometric ratio of cation to anion (Figures S1, S5, and S9, Supplemental Material). Carbon signals (Figures S2, S6, and S10, Supplemental Material) were assigned based on the results of ^13^C spectra, HSQC, and HMBC experiments, which showed the expected ^1^H–^13^C single and multiple bond correlations (Figures S3, S4, S7, S8, S11, and S12, Supplemental Material). Detailed NMR analysis can be found in the Supplementary Material.

Mass peaks corresponding to N_4444_^+^, N_6666_^+^, and P_4444_^+^ were observed in the positive ion mode at *m*/*z* values of 242.2848, 354.4109, and 259.2562, respectively. Analyses of the negative ion mode spectra confirmed the presence of ANS anion in all synthesized compounds. Moreover, there is good agreement between experimental and theoretical *m*/*z* values (Table S1, Supplemental Material). Peaks attributable to starting materials were found in the FT-IR spectra of final products (Figure S13, Supplemental Material), thus providing additional evidence of the successful synthesis of ANS-based GUMBOS.

### Optical Properties of ANS-Based GUMBOS

**Figure 2.**
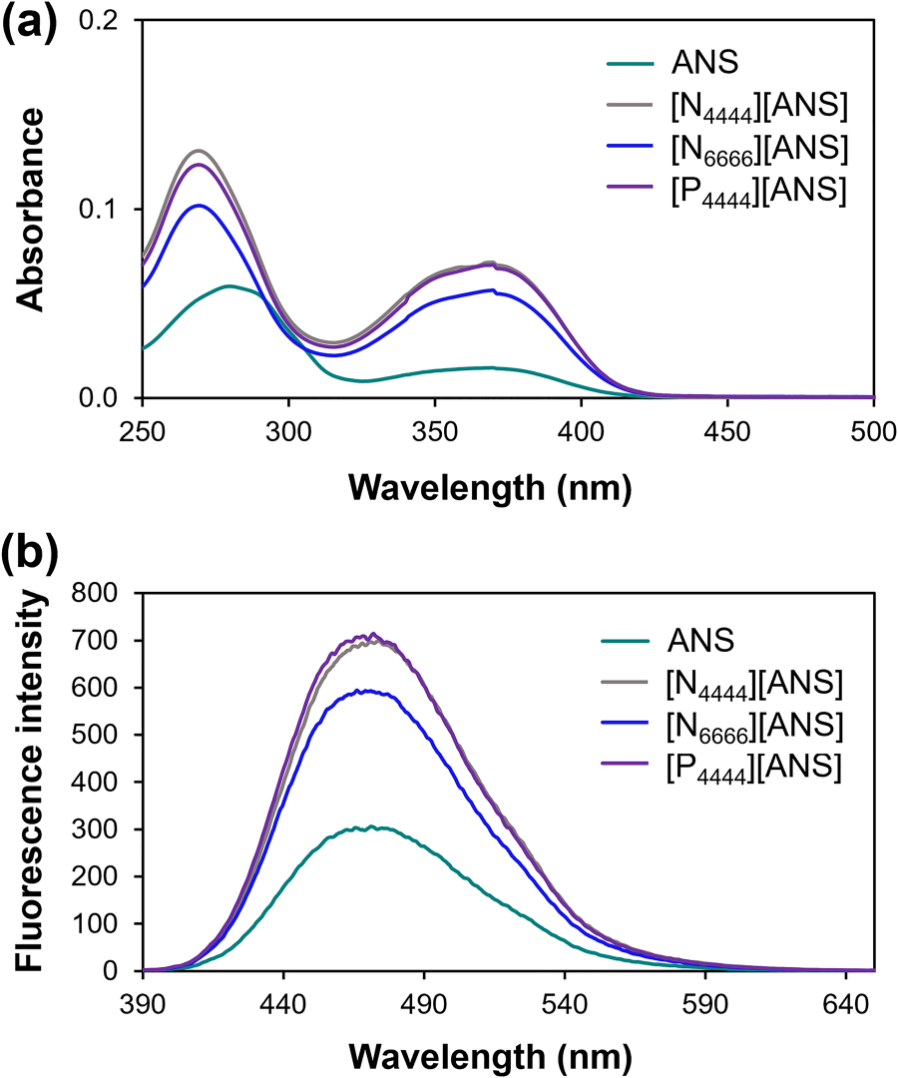
(a) UV–V is and (b) fluorescence spectra ( $\lambda_{\mathrm{exc}}$ = 370 nm) of parent ANS dye and ANS-based GUMBOS at the concentration of 10 µmol L^–1^ in ACN.

Ultraviolet–visible and fluorescence spectroscopies were used to investigate the optical properties of synthesized compounds. The absorption profile of ANS-based GUMBOS (Figure 2a) shows an intense band at 269 nm and a wider, lower intensity, band at 370 nm, which can be assigned to π→π* and n→π* transitions, respectively.^41,42^ Additionally, the band centered at 269 nm is blueshifted by 11 nm as compared to the parent ANS dye. This can be attributed to changes in the microenvironment of ANS (e.g., polarity and hydrophobicity) upon its conversion into GUMBOS.^43–45^ Variations in the extent of hydrogen bonding with solvent molecules, and H-aggregation can also explain the observed shift.^46,47^ According to the exciton theory, H-aggregates are formed by parallel stacking of transition dipole moments and produce a hypsochromic shift in the absorption spectrum with respect to the monomer since only transition to the higher exciton level is allowed.^48,49^ It is also worth noting that charge neutralization of ANS anion by cation incorporation in GUMBOS may lead to a decrease in electrostatic repulsion between dye molecules and subsequent formation of aggregates via π–π interactions.^42,50^

Fluorescence maxima were observed around 470 nm for all compounds (Figure 2b). The higher fluorescence intensity of synthesized GUMBOS in relation to parent ANS can be explained by a reduction in the structural flexibility of ANS dye when in the form of GUMBOS and a decrease in the extent of hydrogen bonding with solvent molecules. Ultimately, this leads to less efficient nonradiative deactivation processes and slower fluorescence decay of excited ANS-GUMBOS.^42,51^

Changes in absorbance and fluorescence intensities are likely the result of structural differences between the cations used in GUMBOS syntheses. For example, an increase in the alkyl chain length of the ammonium cation from four to six carbons (N_4444_^+^ vs. N_6666_^+^) led to a decrease in signal intensity. On the contrary, [N_4444_][ANS] and [P_4444_][ANS] exhibited similar absorbance and fluorescence values, thus suggesting that the cation's central atom (N or P) does not significantly affect the optical properties of synthesized GUMBOS.^52^

### Lipophilicity of the Synthesized Materials

The lipophilicity of ANS-based GUMBOS was evaluated through the determination of micelle/buffer partition coefficients using derivative UV–Vis spectrophotometry. HePC micelles were chosen as membrane models due to the simplicity and rapidity of their preparation, which further eliminates the need for potentially toxic organic solvents (e.g., octanol).^33,53^ In contrast to traditional octanol/water systems, these biomimetic models not only consider hydrophobic interactions, but also hydrogen bonding, electrostatic, and dipole–dipole interactions.^54–56^ Moreover, this method does not require the separation of lipid and aqueous phases, thus leading to better reproducibility. The application of derivative spectrophotometry helps to minimize the effects of light scattering from HePC micelles and improves the resolution of absorption bands.^57,58^ Partition coefficients estimated for parent ANS dye and ANS-based GUMBOS are provided in Table I. Analyses of data show that lipophilicity can be tuned using cation exchange, with increasing order of ANS < [P_4444_][ANS] < [N_4444_][ANS]. Other studies have demonstrated the impact of cations on GUMBOS lipophilicity.^28,59^ The lipophilicity of [N_6666_][ANS] could not be determined due to solubility problems. For this reason, [N_6666_][ANS] GUMBOS was excluded from further analyses.

**Table I.** Partition coefficients (expressed as *K*_p_ and log *D*) of parent ANS and ANS-based GUMBOS in micelles of HePC (pH 7.4, 37 °C). Results are presented as the mean ± standard deviation of three replicates.

Compound	*K*_p_ (L mol^–1^)	log *D* (dimensionless)
ANS	3877 ± 572	3.98 ± 0.06
[N_4444_][ANS]	95 398 ± 12 397	5.37 ± 0.06
[P_4444_][ANS]	36 967 ± 4112	4.96 ± 0.05

### Thermal Stability and Melting Point of ANS-Based GUMBOS

Thermal behaviors of [N_4444_][ANS] and [P_4444_][ANS] GUMBOS were further investigated using TGA. Figure S14 and Table S2 (Supplemental Material) present the weight loss curves and temperatures at which GUMBOS weight was reduced by 50% (*T*_50%_), respectively. Similar to what has been reported for other ionic compounds, the thermal stability of ANS-based GUMBOS was found to be dependent on the cation employed, with [P_4444_][ANS] being the most stable.^52,60,61^

Finally, melting points were determined using a capillary tube method. The lower melting points of GUMBOS as compared to parent ANS dye (Table S2, Supplemental Material) can be attributed to the relatively large size of N_4444_ and P_4444_ cations. Additionally, the similarity of the results obtained for these two GUMBOS can be explained by the length of the cations’ pendant alkyl side chains (e.g., *n *= 4).^30,62,63^

### Protein-Membrane Binding Studies

**Figure 3.**
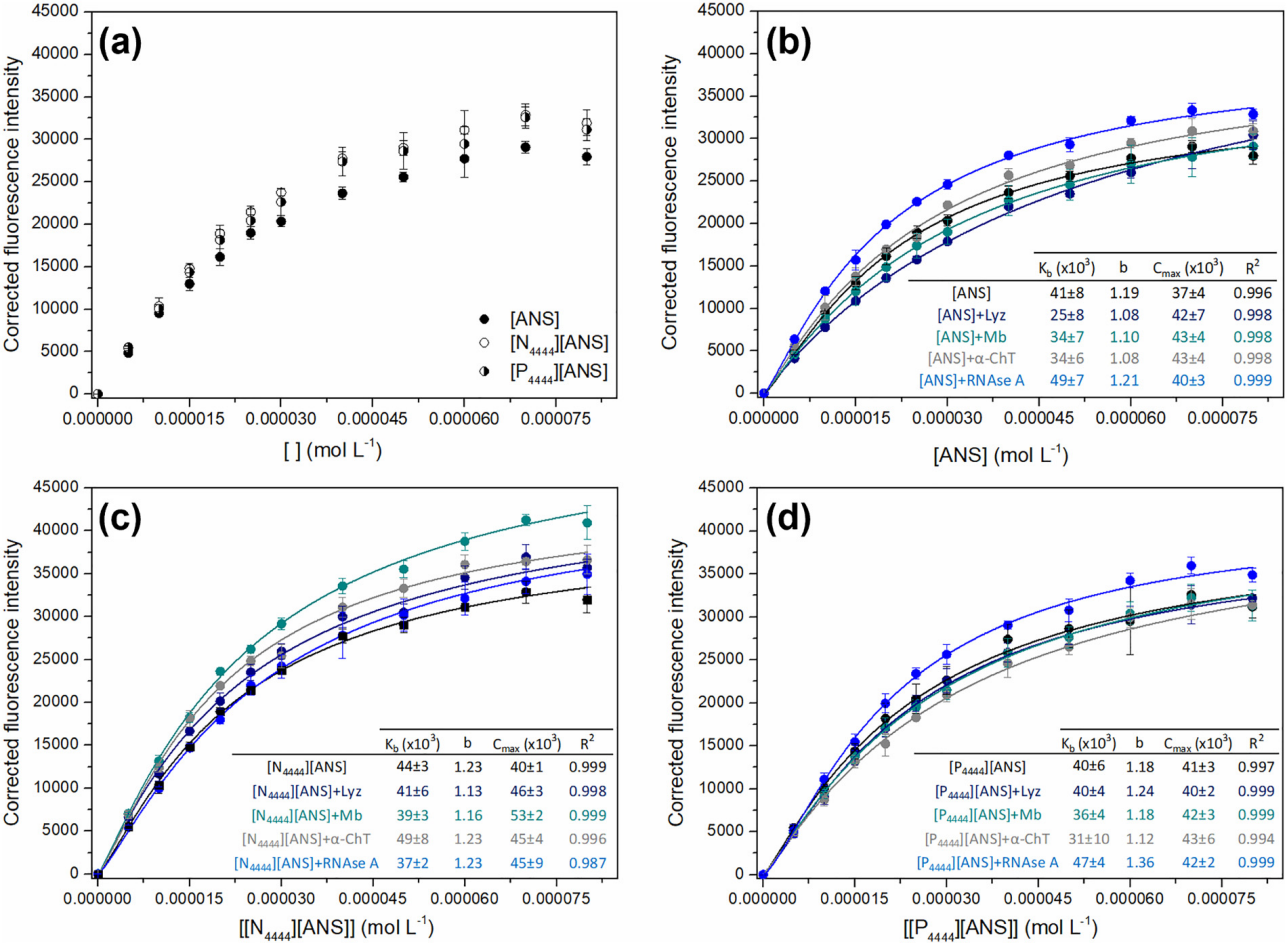
Binding isotherms of ANS, [N_4444_][ANS], and [P_4444_][ANS] in the absence of proteins (a). Binding isotherms of ANS (b), [N_4444_][ANS] (c), and [P_4444_][ANS] (d) in the presence of Lyz, Mb, α-ChT, and RNAse A (20 μg mL^–1^), along with the estimated binding parameters: binding constant (*K*_b_), binding process cooperativity (*b*), maximum fluorescence intensity (*C*_max_), and coefficient of determination (*R*^2^). Results are presented as the mean and standard deviation of at least three independent assays, with lines representing the best fit to Eq. 3.

In order to study protein binding to biomimetic membranes (i.e., DMPC liposomes), a fluorescence titration with parent ANS dye and ANS-based GUMBOS was performed. Fluorescence changes have been used to investigate competition for the same membrane binding sites between proteins and parent ANS as the ANS dye is known to fluoresce when electrostatically bound to phospholipid headgroups.^40,64^ However, it is tempting to hypothesize that the synthesized GUMBOS could form additional interactions with membranes as a result of their tuned features (e.g., lipophilicity and hydrogen-bonding ability), leading to more distinguishable responses upon the addition of proteins.^59^ Binding isotherms were obtained by plotting corrected fluorescence intensity, in the absence (Figure 3a) and presence (Figures 3b–3d) of proteins, as a function of increasing concentrations of parent ANS dye and ANS-based GUMBOS. The resulting curves were then fitted to Eq. 3, thus allowing the calculation of binding constant (*K*_b_), binding process cooperativity (*b*), and maximum fluorescence intensity (*C*_max_). The estimated values for these parameters are presented in embedded tables of Figure 3.

As shown in Figure 3a, parent ANS and ANS-based GUMBOS independently reach saturation within the same concentration range. Despite such findings, GUMBOS exhibited slightly higher fluorescence signals than ANS dye, which could be the result of differences in GUMBOS structures, distinct interactions with liposomes, or even a combination of both. For example, the replacement of the central nitrogen atom by phosphorus should increase the electron density of the cation's protons and lead to weaker electrostatic interactions with DMPC headgroups.^65,66^ Analysis of results further suggests the involvement of hydrophobic interactions in the binding of ANS-GUMBOS to lipid membranes. In fact, a direct relationship between compounds’ lipophilicity and fluorescence intensity was demonstrated. The exact mechanism of interaction still needs to be clarified, but it is reasonable to presume that GUMBOS are located deeper in the liposome than parent ANS, with the cation alkyl chains inserted into the lipid membrane. Moreover, the obtained results show that it is possible to tune sensitivity by converting ANS into GUMBOS, similar to what has been found in previous works.^52,67^

It is worth noting that each protein (i.e., Lyz, Mb, α-ChT, and RNAse A) produced distinct fluorescence responses with parent ANS, [N_4444_][ANS], and [P_4444_][ANS] (Figures 3b to 3d). Moreover, different fluorescence signals were obtained for the same protein when using these three probes. These findings suggest that membrane binding is affected by protein properties, such as surface hydrophobicity, which further corroborates previous reports.^68,69^ For example, there was a decrease in the *K*_b_ value determined for [N_4444_][ANS] in the presence of RNAse A (the most hydrophilic among selected proteins), revealing low affinity of this protein for GUMBOS binding sites.^14,70^ In turn, an observed increase in *K*_b_ when proteins are present suggests higher dissociation of fluorescent probes and, consequently, weaker membrane binding. The relative maintenance of values for *b* and *C*_max_ further indicates that proteins do not change binding rates of parent ANS or ANS-based GUMBOS, but rather hinders their access to membranes through a competitive mechanism.^40^

## Conclusion

Herein, three novel ANS-based GUMBOS ([N_4444_][ANS], [N_6666_][ANS], and [P_4444_][ANS]) were successfully synthesized from ANS dye using simple ion metatheses reactions, and their potential as fluorescent probes for study of protein-membrane binding was investigated. It was found that [N_4444_][ANS] and [P_4444_][ANS] display enhanced optical properties when compared to parent ANS and [N_6666_][ANS]. These two GUMBOS also showed improved lipophilicity as well as good thermal stability, which is why they were selected for subsequent binding studies. Although membrane saturation occurred at the same concentration range for [N_4444_][ANS], [P_4444_][ANS], and ANS, slightly enhanced fluorescence signals were observed for synthesized GUMBOS as compared to parent ANS dye. Additionally, different responses were obtained for each probe upon the addition of proteins, which demonstrates the impact of protein properties (e.g., surface hydrophobicity) on their binding ability to lipid membranes. No major changes were observed in the binding process cooperativity (*b*) or maximum fluorescence intensity (*C*_max_), which suggests that proteins and ANS-based GUMBOS synthesized herein compete for the same binding sites on membranes. Overall, these findings provide valuable information for the design of ANS-based GUMBOS with improved properties as probes. Moreover, this GUMBOS-based strategy is operationally simple and requires relatively small concentrations of reagents, which makes it attractive for high-throughput analyses. As part of future work, it is also planned to use a wider range of cations in the synthesis of ANS-GUMBOS (e.g., distinct alkyl side chain lengths) and investigate their effects on tuning ANS properties.

## Supplemental Material

sj-docx-1-asp-10.1177_00037028241249768 - Supplemental material for Studies of Protein Binding to Biomimetic Membranes Using a Group of Uniform Materials Based on Organic Salts Derived From 8-Anilino-1-naphthalenesulfonic Acid Supplemental material, sj-docx-1-asp-10.1177_00037028241249768 for Studies of Protein Binding to Biomimetic Membranes Using a Group of Uniform Materials Based on Organic Salts Derived From 8-Anilino-1-naphthalenesulfonic Acid by Ana M.O. Azevedo, Cláudia Nunes, Tânia Moniz, Rocío L. Pérez, Caitlan E. Ayala, Maria Rangel, Salette Reis, João L.M. Santos, Isiah M. Warner and M. Lúcia M.F.S. Saraiva in Applied Spectroscopy
