# Supplementary material for: Studies of Protein Binding to Biomimetic Membranes Using a Group of Uniform Materials Based on Organic Salts Derived From 8-Anilino-1-naphthalenesulfonic Acid
Source: Appl Spectrosc. 2024 May 15;78(8):806–14. doi: 10.1177/00037028241249768 (PMC11340245; doi:10.1177/00037028241249768)
Supplement: sj-docx-1-asp-10.1177_00037028241249768 - Supplemental material for Studies of Protein Binding to Biomimetic Membranes Using a Group of Uniform Materials Based on Organic Salts Derived From 8-Anilino-1-naphthalenesulfonic Acid [file sj-docx-1-asp-10.1177_00037028241249768.docx]

**Supplementary Material**

**Studies of Protein Binding to Biomimetic Membranes Using a Group of Uniform Materials Based on Organic Salts Derived from 8-Anilino-1-naphthalenesulfonic Acid**

Ana M.O. Azevedo ^1^, Cláudia Nunes ^1,^, Tânia Moniz ^2,3^, Rocío L. Pérez ^4,5^, Caitlan E. Ayala ^4^, Maria Rangel ^3^, Salette Reis ^1^, João L.M. Santos ^1^, Isiah M. Warner ^4^, and M. Lúcia M.F.S. Saraiva ^1^

^1^ LAQV, REQUIMTE, Departamento de Ciências Químicas, Faculdade de Farmácia, Universidade do Porto, Rua Jorge Viterbo Ferreira 228, 4050-313 Porto, Portugal

^2^ LAQV, REQUIMTE, Departamento de Química e Bioquímica, Faculdade de Ciências, Universidade do Porto, Rua Campo Alegre s/n, 4169-007 Porto, Portugal

^3^ LAQV, REQUIMTE, Instituto de Ciências Biomédicas Abel Salazar, Universidade do Porto, Rua Jorge Viterbo Ferreira 228, 4050-313 Porto, Portugal

^4^ Department of Chemistry, Louisiana State University, Baton Rouge, Louisiana 70803, United States

^5^ Department of Chemistry and Biochemistry, Georgia Southern University, Statesboro, Georgia 30458, United States

**Corresponding author:**

M. Lúcia M.F.S. Saraiva, LAQV, REQUIMTE, Departamento de Ciências Químicas, Faculdade de Farmácia, Universidade do Porto, Rua Jorge Viterbo Ferreira 228, 4050-313 Porto, Portugal.

Email: lsaraiva@ff.up.pt

# **NMR characterization of ANS-based GUMBOS**

*[N_4444_][ANS]*

^1^H NMR (400.15 MHz, CD_3_OD, ppm): δ 8.38 (dd, *J* 7.4, 1.5, 1H, H2); 7.91 (dd, *J* 8.2, 1.4, 1H, H4); 7.58 (dd, *J* 7.7, 1.4, 1H, H7); 7.44 (dd, *J* 8.0, 1.4, 1H, H5); 7.41–7.35 (m, 2H, H3 and H6); 7.20–7.15 (m, 4H, 2 x H10 and 2x H11); 6.81–6.76 (m, 1H, H12); 3.18–3.14 (m, 4 x 2H, -NCH_2_CH_2_CH_2_CH_3_); 1.63–1.55 (m, 4 x 2H, -NCH_2_CH_2_CH_2_CH_3_); 1.40–1.31 (m, 4 x 2H, -NCH_2_CH_2_CH_2_CH_3_); 0.98 (t, *J* 7.4, 4 x 3H, -NCH_2_CH_2_CH_2_CH_3_). ^13^C NMR (100.62 MHz, CD_3_OD, ppm): δ 146.2 (C9); 141.7 (C8); 141.1 (C1); 138.8 (C4a); 134.0 (C4); 129.9 (C11); 128.7 (C2); 127.2 (C6); 124.8 (C3); 124.0 (C8a); 122.6 (C5); 120.7 (C12); 118.5 (C10); 117.6 (C7); 59.4 (-NCH_2_CH_2_CH_2_CH_3_); 24.8 (-NCH_2_CH_2_CH_2_CH_3_); 20.7 (-NCH_2_CH_2_CH_2_CH_3_); 13.9 (-NCH_2_CH_2_CH_2_CH_3_).

*[N_6666_][ANS]*

^1^H NMR (400.15 MHz, CD_3_OD, ppm): δ 8.38 (dd, *J* 7.4, 1.5, 1H, H2); 7.90 (dd, *J* 8.2, 1.4, 1H, H4); 7.57 (dd, *J* 7.7, 1.4, 1H, H7); 7.44 (dd, *J* 8.0, 1.4, 1H, H5); 7.38 (q, 2H, H3 and H6); 7.20–7.15 (m, 4H, 2 x H10 and 2x H11); 6.80–6.76 (m, 1H, H12); 3.20 (quint, *J* 5.1, 3.4, 4 x 2H, -NCH_2_CH_2_CH_2_CH_2_CH_2_CH_3_); 1.64 (quint, *J* 7.4, 8.0, 4 x 2H, -NCH_2_CH_2_CH_2_CH_2_CH_2_CH_3_); 1.34–1.39 (m, 12 x 2H, -NCH_2_CH_2_CH_2_CH_2_CH_2_CH_3_); 0.93 (quint, *J* 7.4, 8.0, 4 x 3H, -NCH_2_CH_2_CH_2_CH_2_CH_2_CH_3_). ^13^C NMR (100.62 MHz, CD_3_OD, ppm): δ 146.2 (C9); 141.1 and 141.6 (C1 + C8); 138.8 (C4a); 134.1 (C4); 130.0 (C11); 128.7 (C2); 127.2 (C6); 124.8 (C3); 124.0 (C8a); 122.6 (C5); 120.7 (C12); 118.5 (C10); 117.7 (C7); 59.6 (-NCH_2_CH_2_CH_2_CH_2_CH_2_CH_3_); 32.4 (-NCH_2_CH_2_CH_2_CH_2_CH_2_CH_3_); 27.0 (-NCH_2_CH_2_CH_2_CH_2_CH_2_CH_3_); 23.5 (-NCH_2_CH_2_CH_2_CH_2_CH_2_CH_3_); 22.7 (-NCH_2_CH_2_CH_2_CH_2_CH_2_CH_3_); 14.3 (-NCH_2_CH_2_CH_2_CH_2_CH_2_CH_3_).

*[P_4444_][ANS]*

^1^H NMR (400.15 MHz, CD_3_OD, ppm): 8.38 (dd, *J* 7.4, 1.5, 1H, H2); 7.90 (dd, *J* 8.2, 1.5, 1H, H4); 7.57 (dd, *J* 7.6, 1.2, 1H, H7); 7.44 (dd, *J* 8.1, 1.5, 1H, H5); 7.40–7.35 (m, 2H, H3 and H6); 7.20–7.15 (m, 4H, 2 x H10 and 2x H11); 6.80–6.76 (m, 1H, H12); 2.15 (bs, 4 x 2H, -PCH_2_CH_2_CH_2_CH_3_); 1.50 (bs, 4 x 2H, -PCH_2_CH_2_CH_2_CH_3_ and 4 x 2H, -PCH_2_CH_2_CH_2_CH_3_); 0.97 (bs, 4 x 3H, -PCH_2_CH_2_CH_2_CH_3_). ^13^C NMR (100.62 MHz, CD_3_OD, ppm): δ 146.2 (C9); 141.7 (C8); 141.2 (C1); 138.8 (C4a); 134.1 (C4); 130.0 (C11); 128.7 (C2); 127.2 (C6); 124.8 (C3); 124.1 (C8a); 122.6 (C5); 120.7 (C12); 118.5 (C10); 117.6 (C7); 25.0–24.3 (-PCH_2_CH_2_CH_2_CH_3_); 19.3–18.9 (-PCH_2_CH_2_CH_2_CH_3_); 13.6 (-PCH_2_CH_2_CH_2_CH_3_).

Analysis of ^1^H NMR spectra of [N_4444_][ANS] (Fig. S1) revealed two sets of signals: one corresponding to the aliphatic protons of N_4444_^+^ and the other to the aromatic protons of ANS anion. Resonance signals of methyl protons at the end of the cation alkyl side chains appeared at 0.98 ppm and aliphatic ethylene protons were seen between 1.63 and 1.31 ppm. Protons registered at *δ* = 3.18–3.14 ppm were assigned to the ethylene groups directly bound to the central nitrogen atom. The first set of aromatic protons corresponds to the phenyl ring of ANS^-^ and signals in the low field area of the ^1^H spectrum were attributed to protons of the naphthalene group. The recorded signals were in good agreement with assignments reported in the literature for ANS.^1,2^


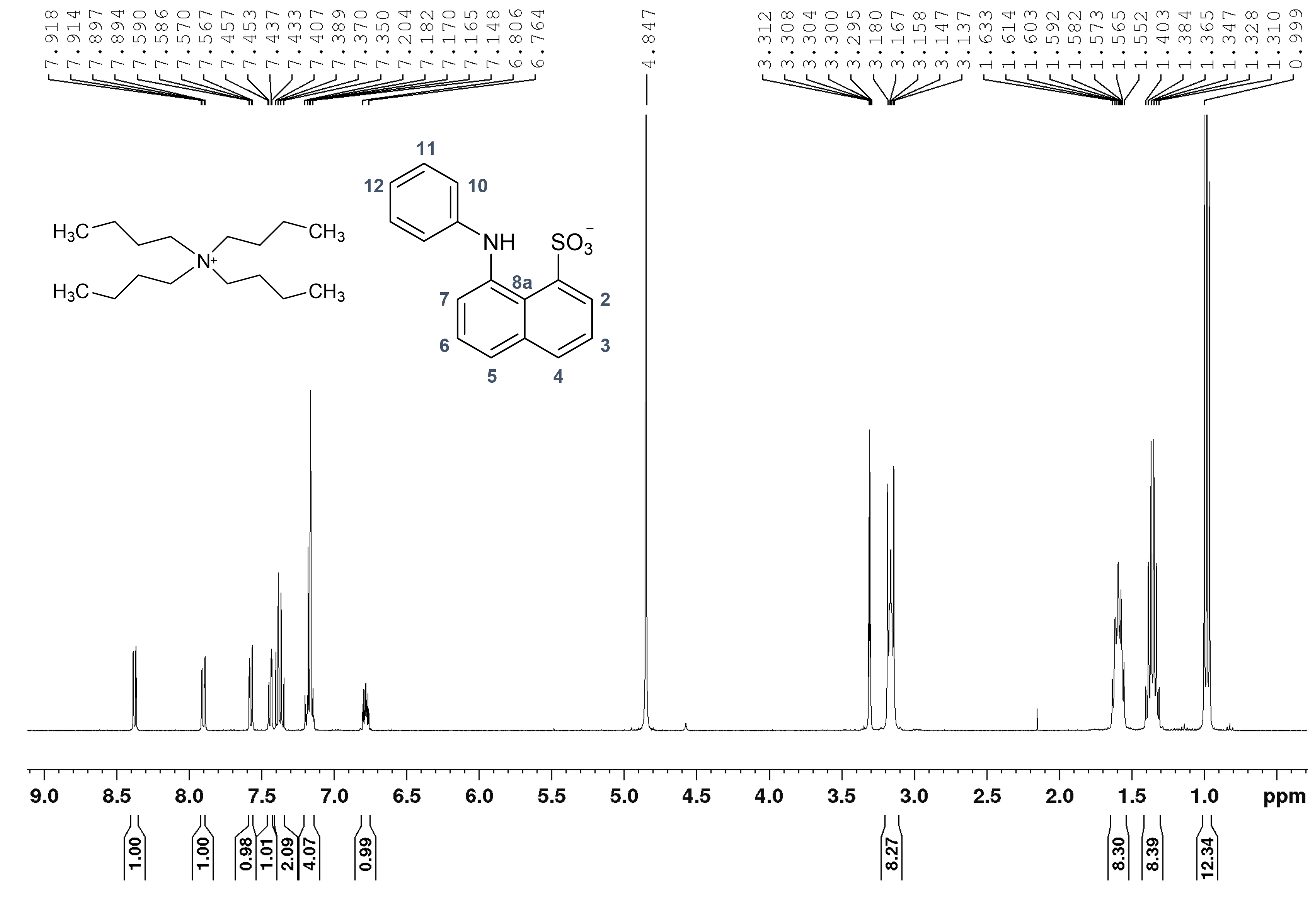


**Figure S1.** ^1^H NMR spectrum of [N_4444_][ANS].

Carbon signals in the range of 59.4–13.9 ppm (Fig. S2) were directly assigned as they showed HSQC correlations with the respective protons in the cation (Fig. S3). Resonance signals of carbons from ANS^-^ were attributed based on their correlation with protons in HSQC and HMBC spectra (Figs. S3 and S4, respectively). Experimental chemical shift values were similar to the expected ones.^1,2^


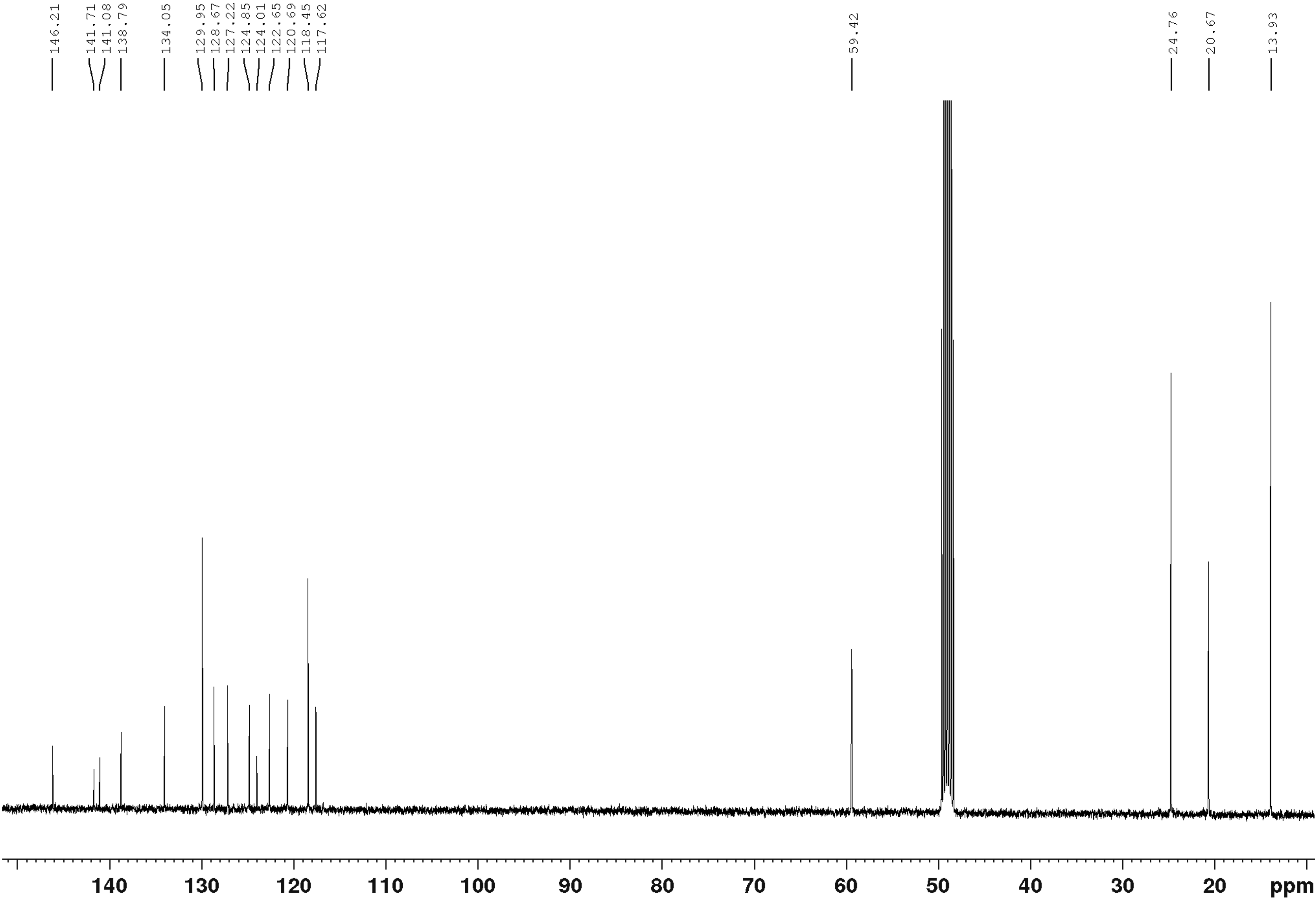


**Figure S2.** ^13^C NMR spectrum of [N_4444_][ANS] GUMBOS.


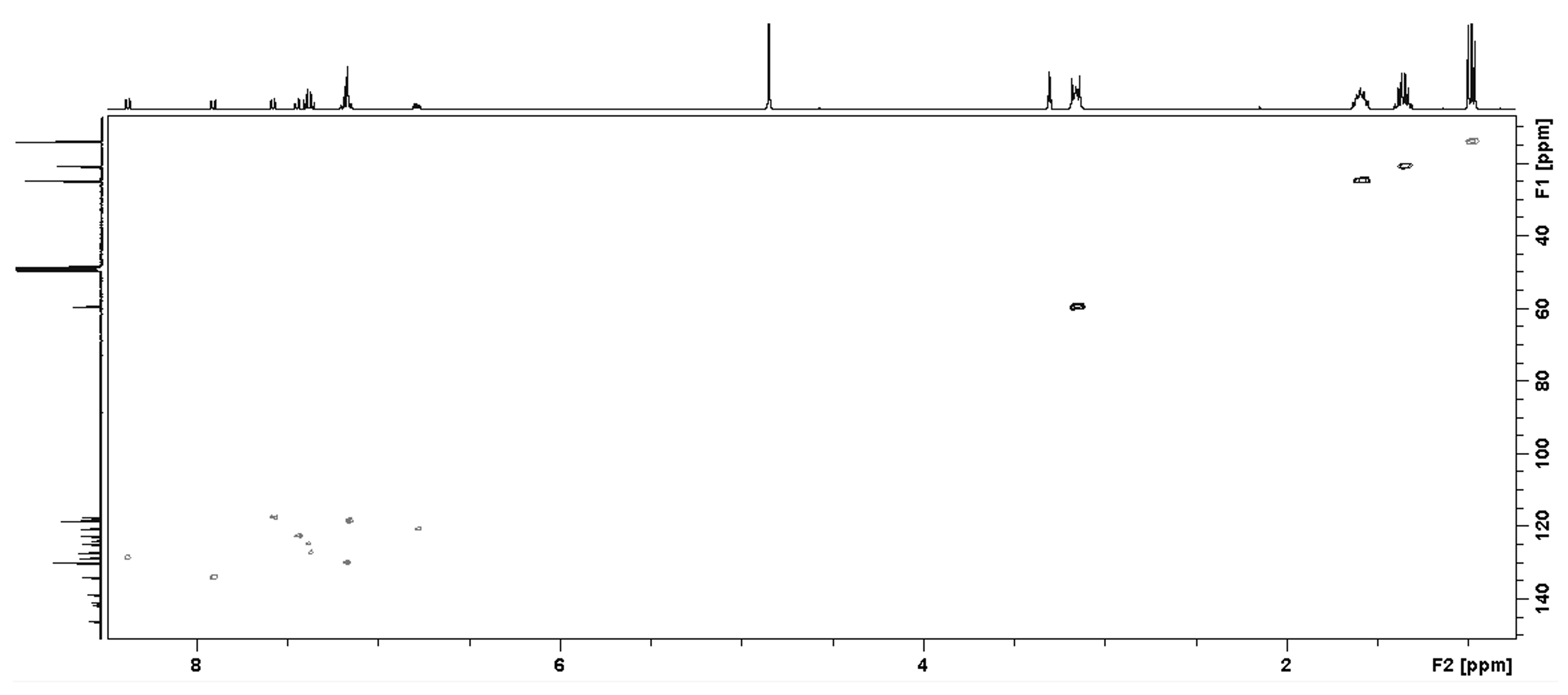


**Figure S3.** HSQC spectrum of [N_4444_][ANS].


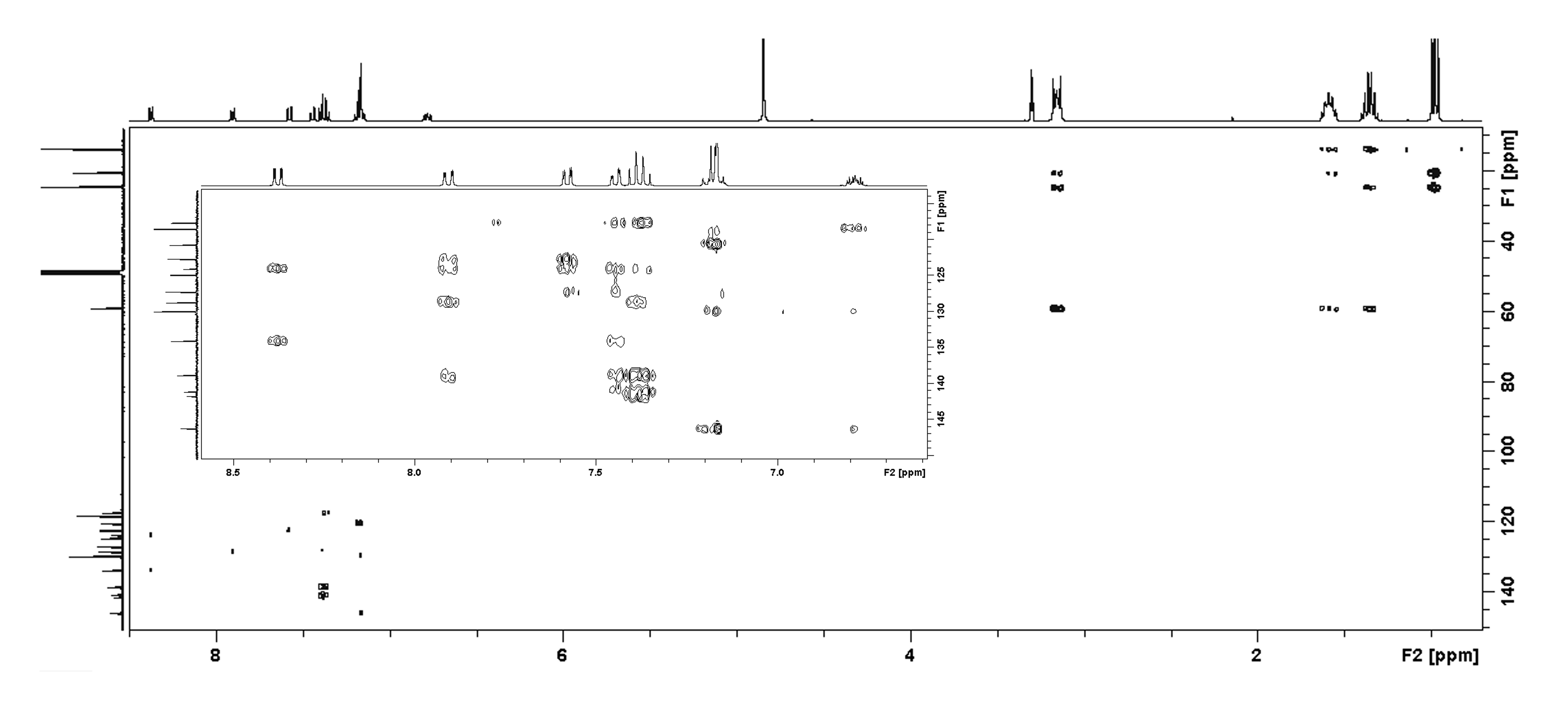


**Figure S4.** HMBC spectrum of [N_4444_][ANS] and respective inset.

The ^1^H and ^13^C signals of [N_6666_][ANS] GUMBOS (Figs. S5 and S6, respectively) were quite similar to those found for [N_4444_][ANS], which is not surprising due to the chemical resemblance between these compounds, with the only difference being the length of alkyl side chains (six *versus* four carbons).


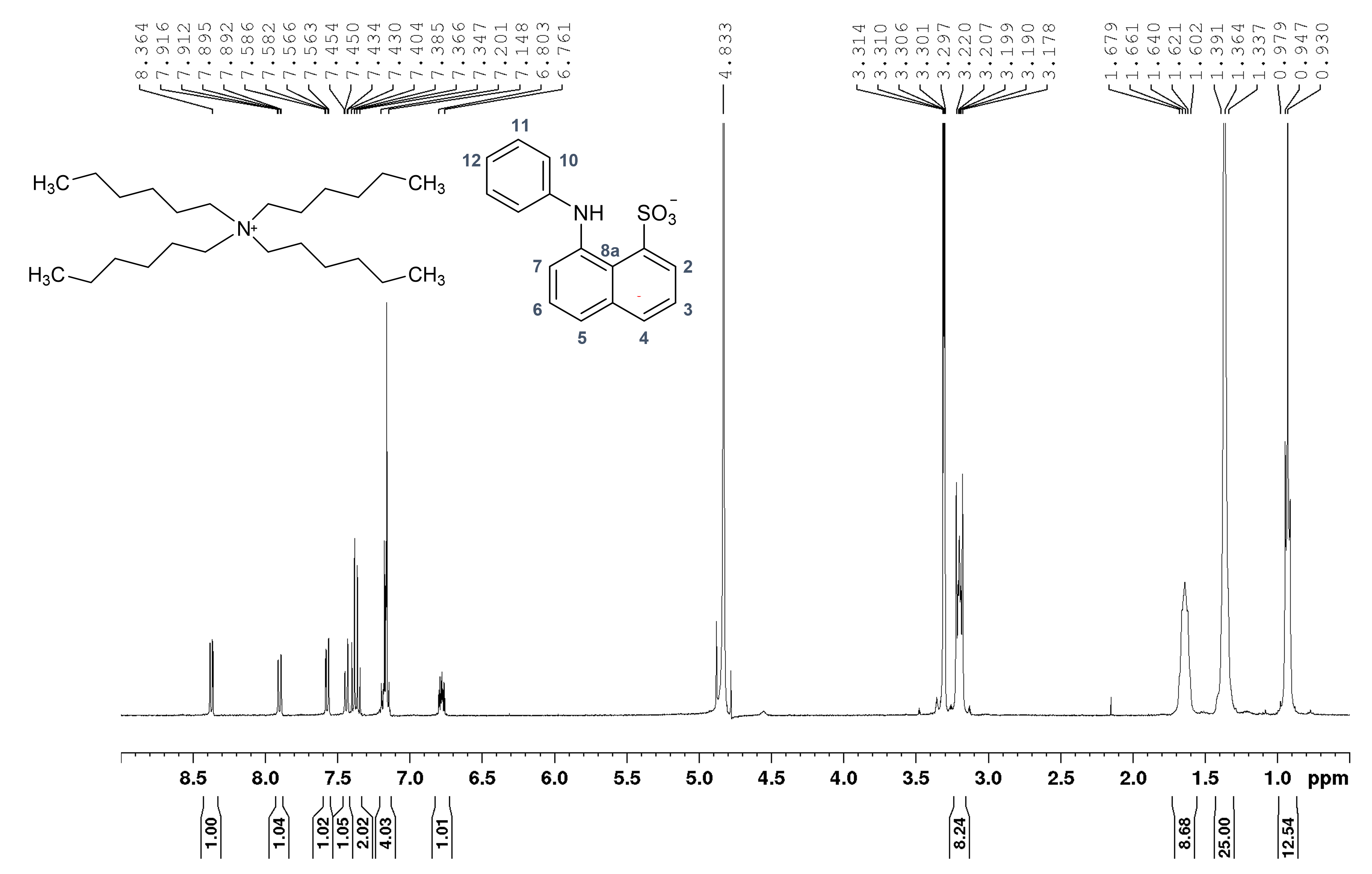


**Figure S5.** ^1^H spectrum of [N_6666_][ANS] GUMBOS.


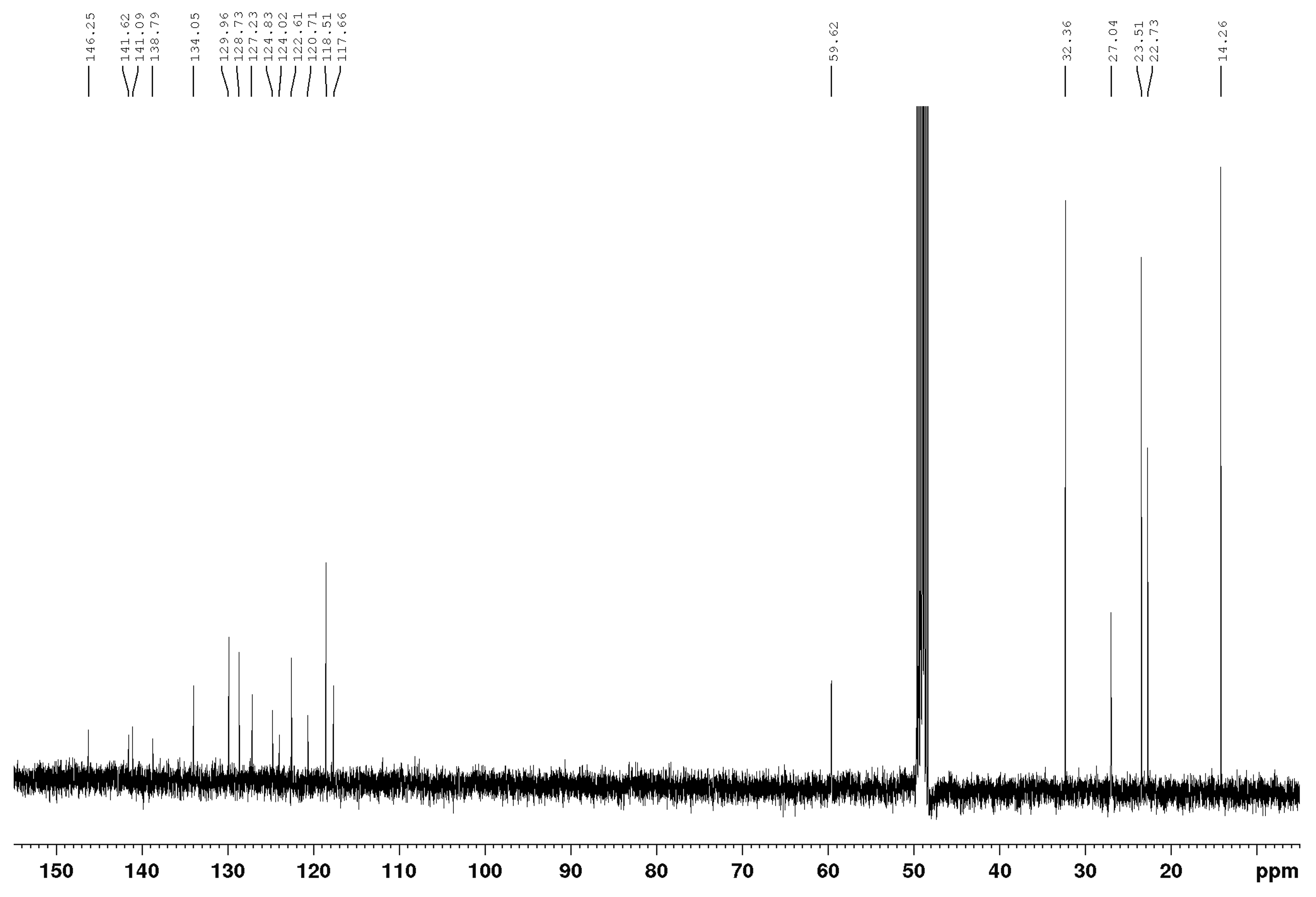


**Figure S6.** ^13^C spectrum of [N_6666_][ANS] in CD_3_OD.

Aliphatic protons and carbons appeared at 3.20–0.93 and 59.6–14.3 ppm, respectively. Carbons were assigned based on their HSQC and HMBC correlations (Figs. S7 and S8, respectively). Additionally, the aromatic peaks of ANS anion were found to be virtually equal to those of [N_4444_][ANS], and in line with previously reported values.^1,2^


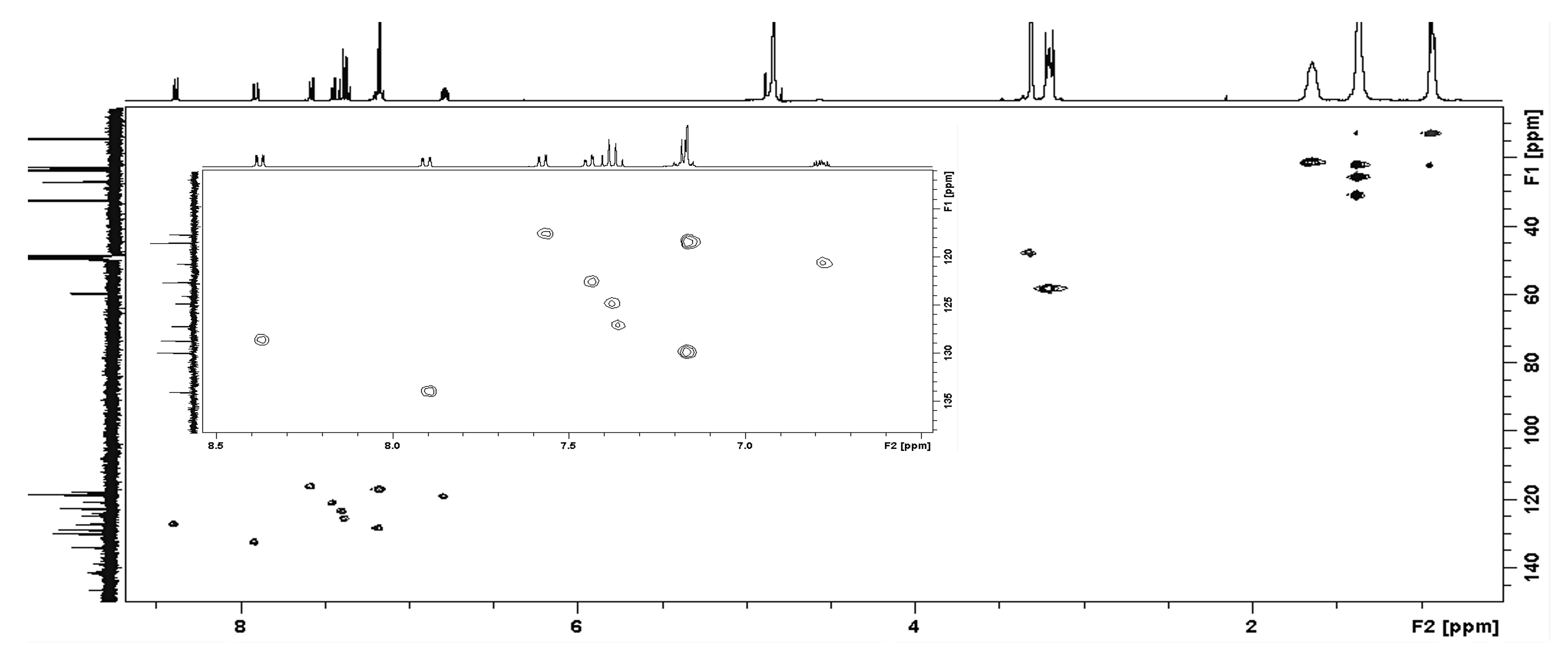


**Figure S7.** HSQC spectrum of [N_6666_][ANS] GUMBOS and respective inset.


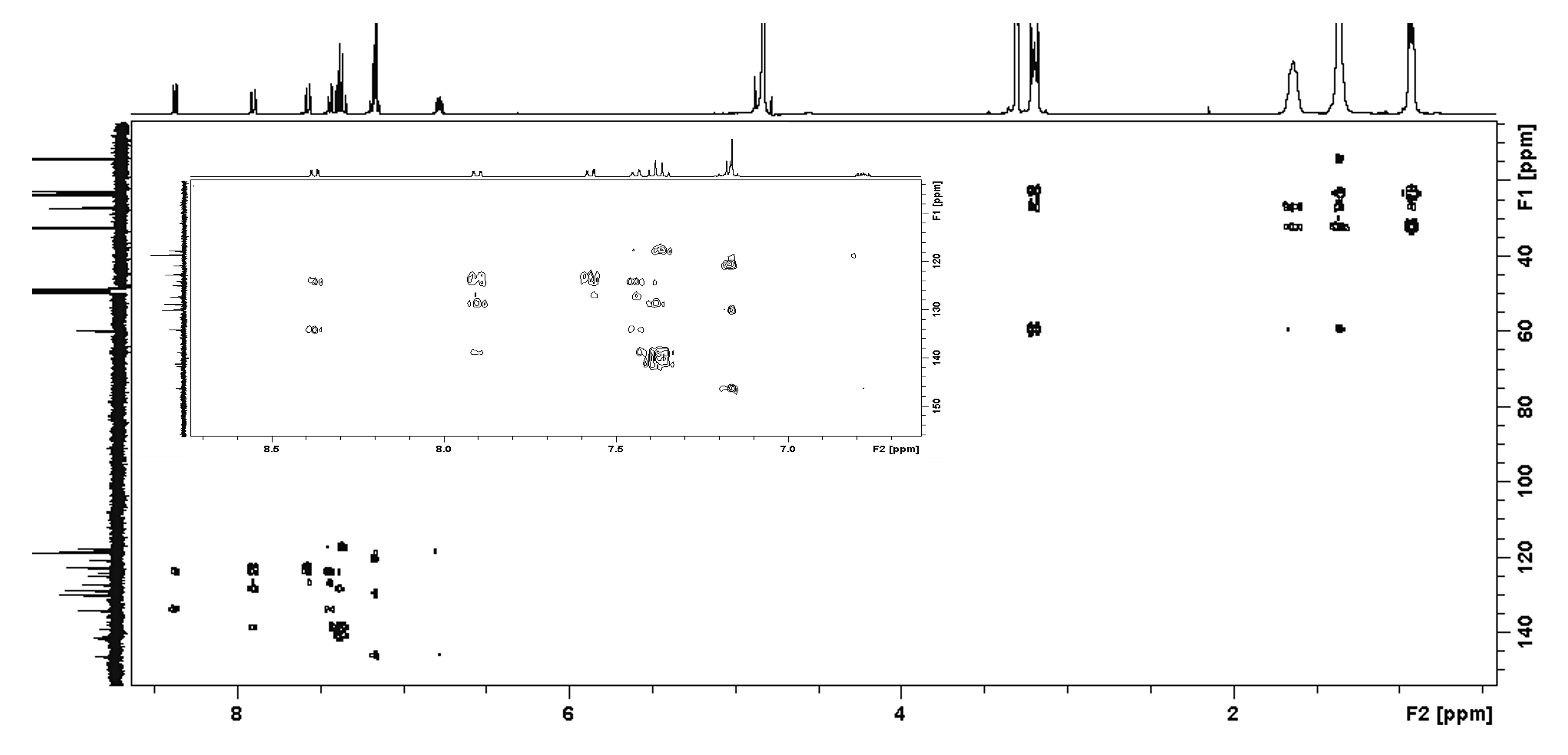


**Figure S8.** HMBC spectrum of [N_6666_][ANS] and respective inset.

Regarding [P_4444_][ANS] GUMBOS, the terminal -CH_3_ and -CH_2_- protons of cation alkyl side chains appeared as broad singlets at *δ* = 0.97 and 2.15–1.50 ppm, respectively (Fig. S9). These signals were found at lower chemical shift values than those for [N_4444_][ANS]. Moreover, resonance signals of protons in the middle of the alkyl chain (-PCH_2_CH_2_CH_2_CH_3_) have similar neighboring, thus appearing as a single peak in the ^1^H spectrum. The stronger electron-withdrawing effect of nitrogen atom compared to phosphorus in the cation can explain the different chemical shifts of -NCH_2_- protons *versus* -PCH_2_- (from 3.18–3.14 to 2.15 ppm, respectively). Similar to [N_4444_][ANS] GUMBOS, aromatic peaks were assigned to the protons of ANS anion. Chemical shifts were very close and in line with reported values.^1,2^


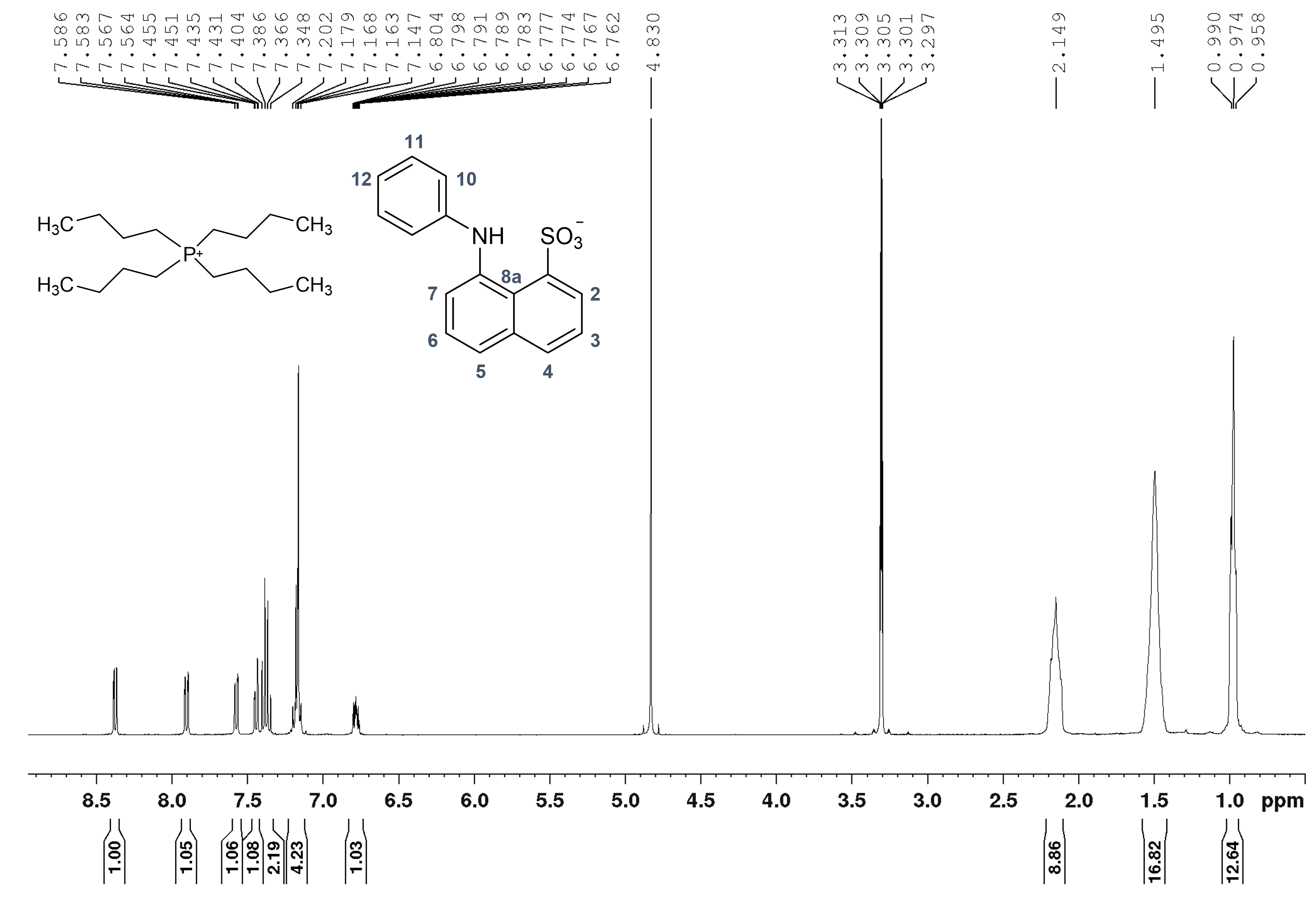


**Figure S9.** ^1^H spectrum of [P_4444_][ANS].

Regarding the assignment of carbons present in [P_4444_][ANS] GUMBOS (Fig. S10), the HSQC spectrum (Fig. S11) showed peaks from 25.0 to 13.6 ppm, which were correlated with signals attributed to protons of the aliphatic chains of P_4444_^+^. Once again, carbons in the middle of the chain (-PCH_2_CH_2_CH_2_CH_3_) had similar neighboring and appeared closer in the ^13^C spectrum. The chemical shift is significantly lower than that verified for similar carbons in the [N_4444_][ANS] GUMBOS. For example, the signal assigned to carbons of -PCH_2_- groups is shifted to a higher field, varying from 59.4 ppm in [N_4444_][ANS] to approximately 19 ppm in [P_4444_][ANS]. This difference can be justified by the influence of cation’s central atom (N or P).


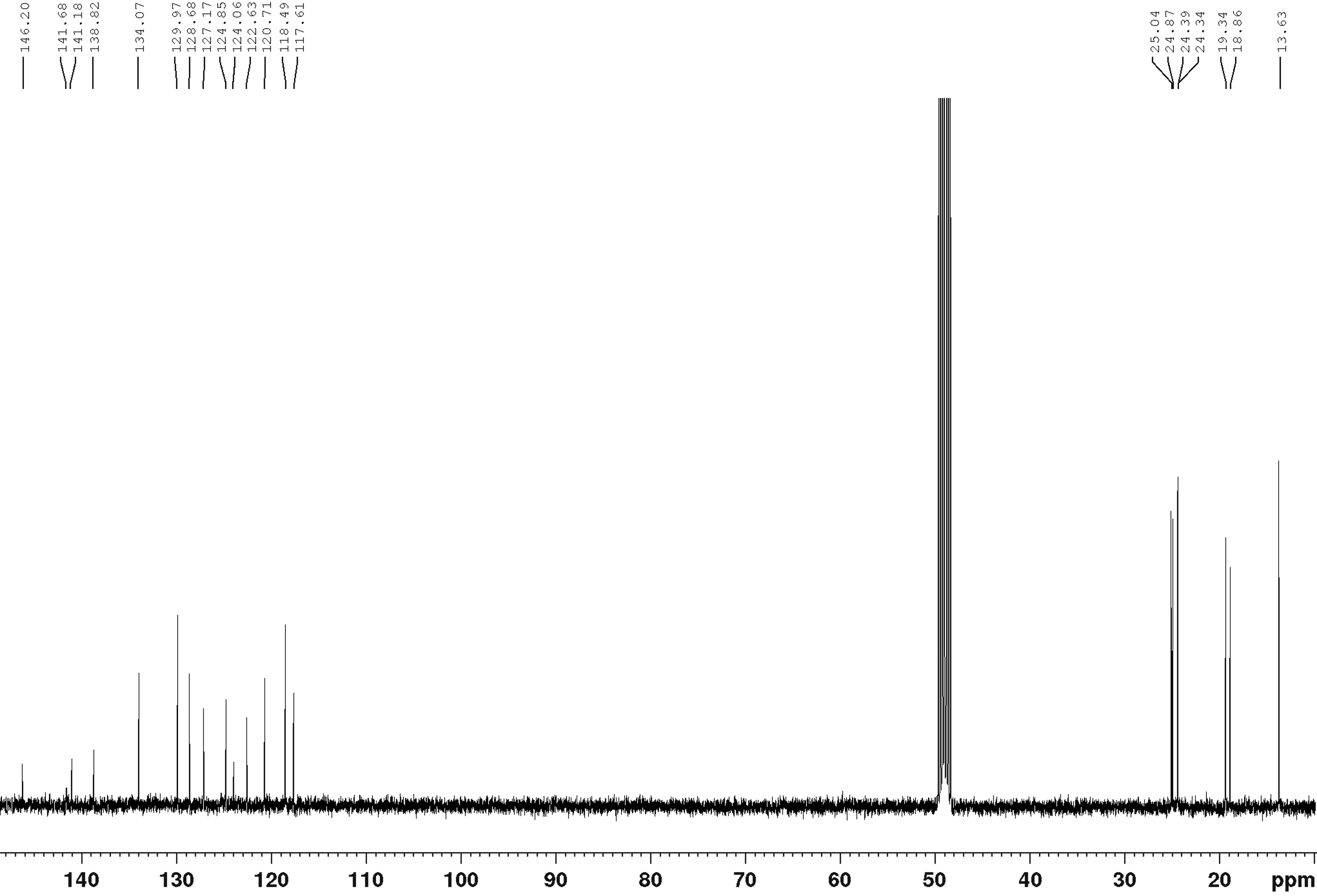


**Figure S10.** ^13^C spectrum of [P_4444_][ANS] GUMBOS.

Analysis of HSQC and HMBC spectra (Figs. S11 and S12) allowed the unequivocal assignment of peaks attributed to carbons present in the ANS anion, which appeared in the low-field region. Their chemical shift values were in good agreement with the values described in the literature.^1,2^


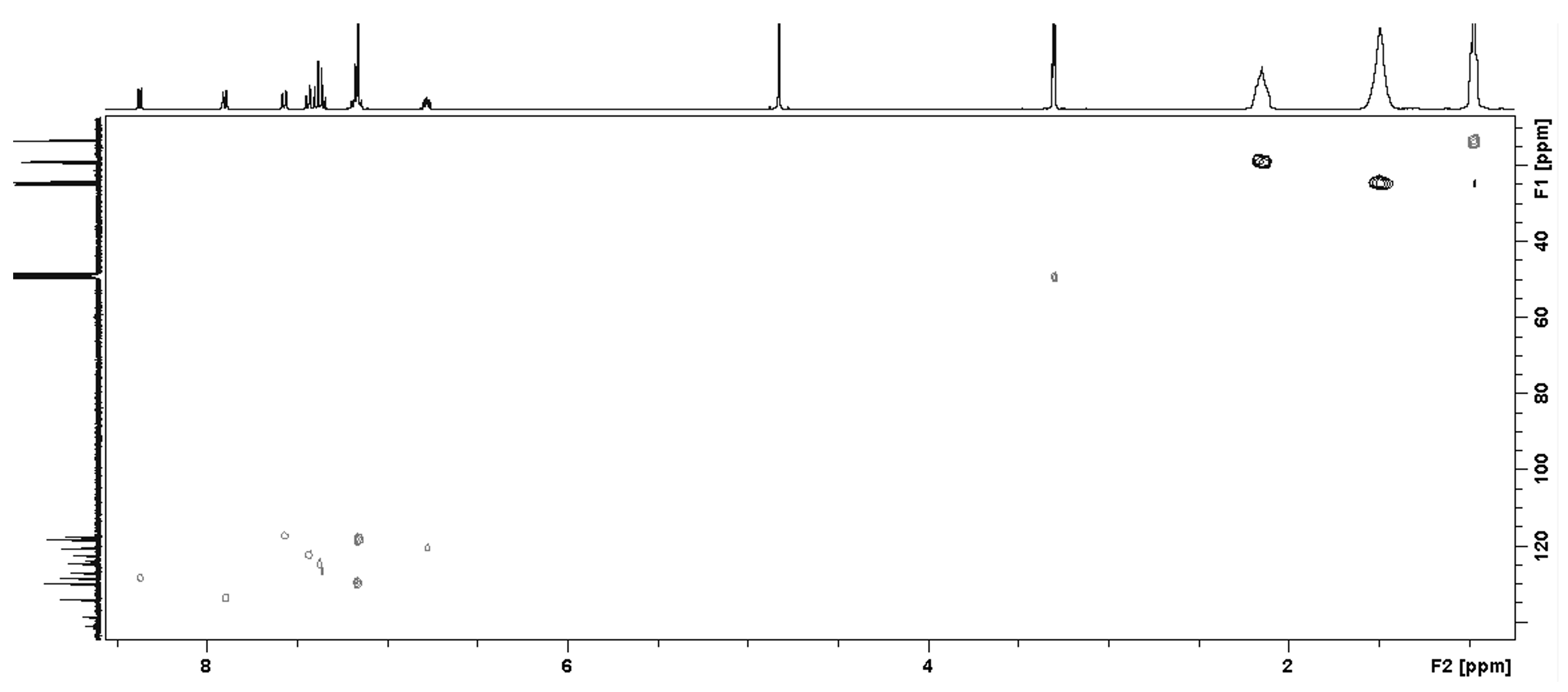


**Figure S11.** HSQC spectrum of [P_4444_][ANS].


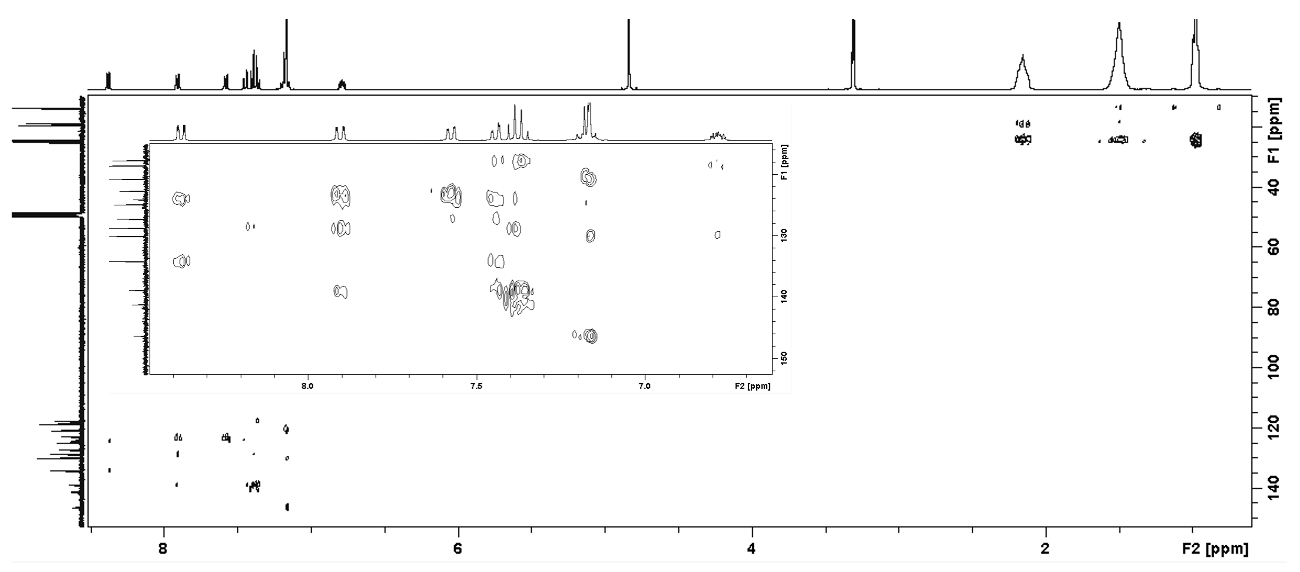


**Figure S12.** HMBC spectrum of [P_4444_][ANS] and respective inset.


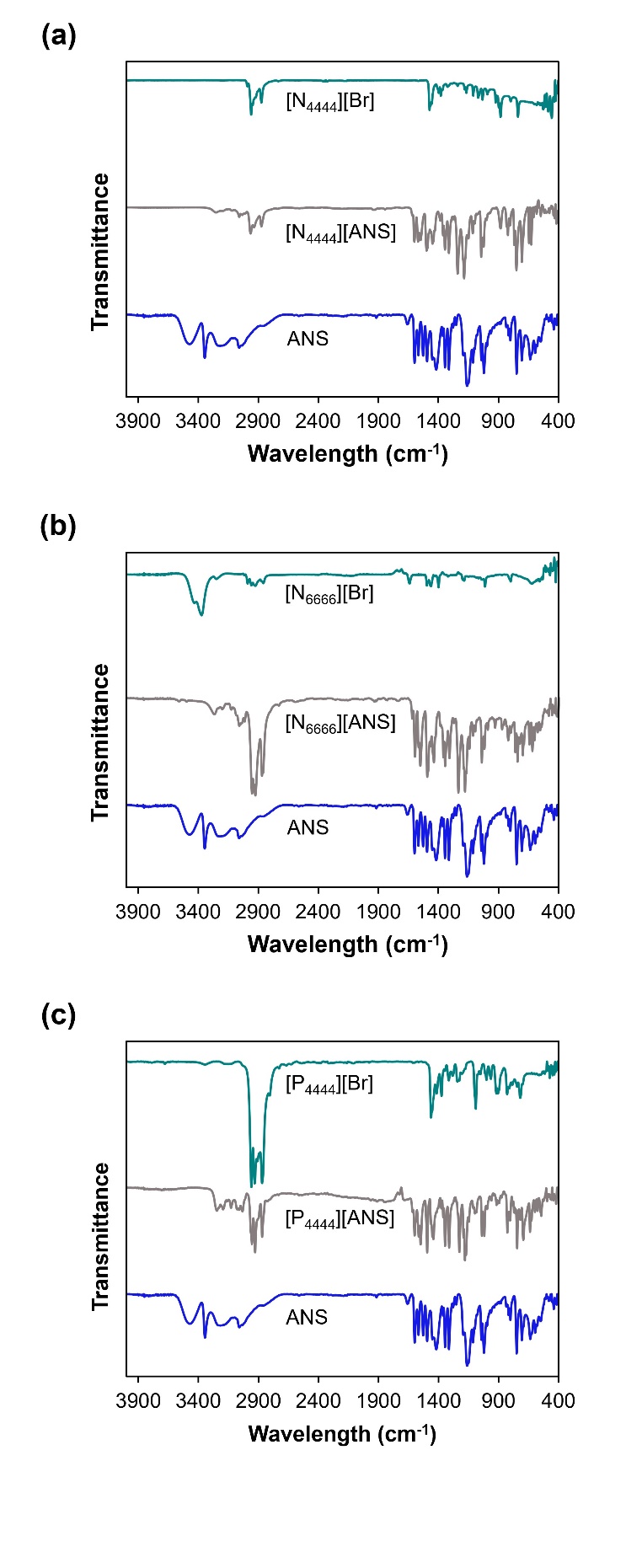


**Figure S13.** Overlay of FTIR spectra of starting materials (green and blue) and resulting GUMBOS (grey): (a) [N_4444_][ANS], (b) [N_6666_][ANS], and (c) [P_4444_][ANS].


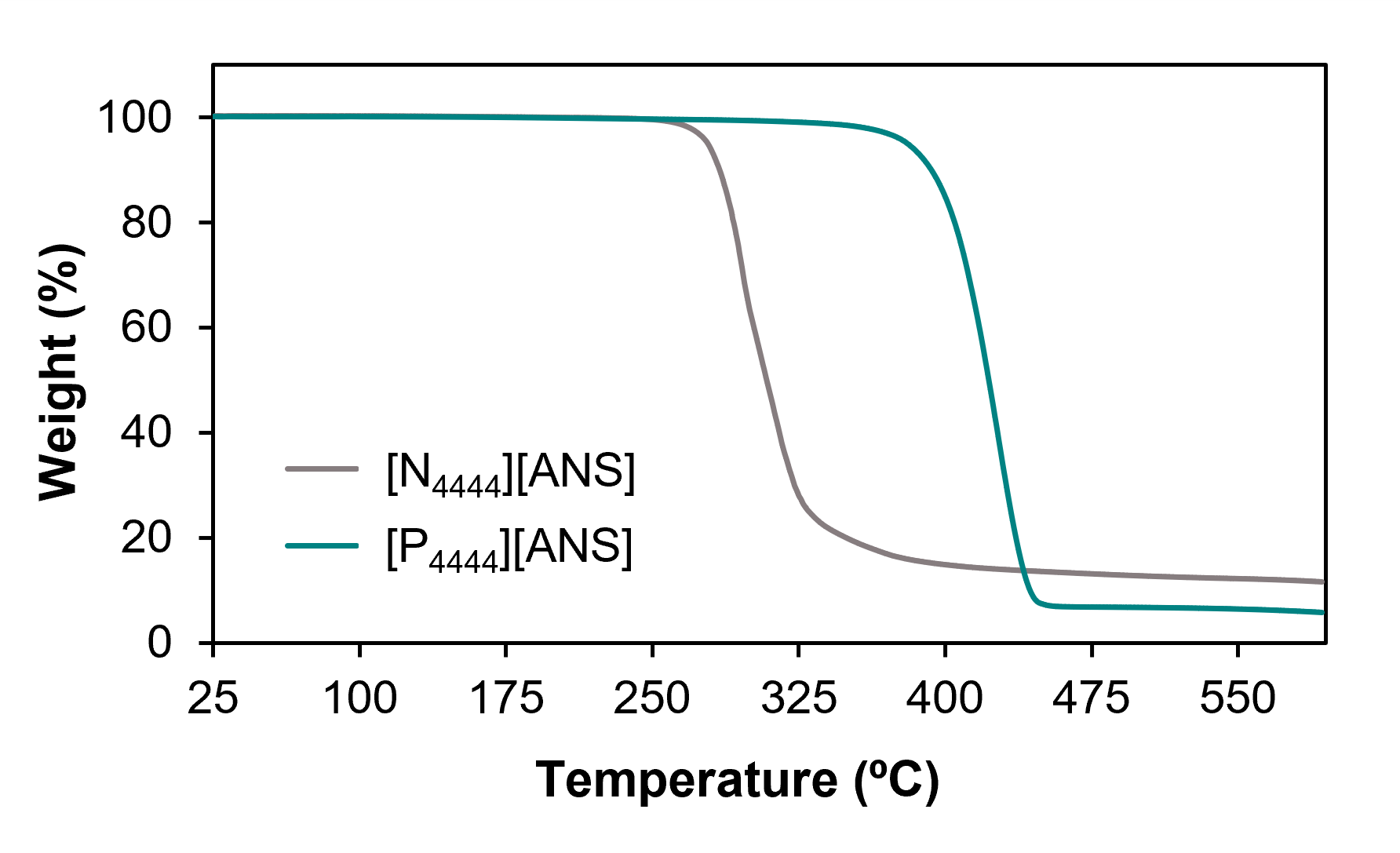


**Figure S14.** TGA spectra of ANS-based GUMBOS.

| **GUMBOS** | **Ionic formula** | | **ESI-MS (positive mode)** | | **ESI-MS (negative mode)** | |
| --- | --- | --- | --- | --- | --- | --- |
|  | Cation | Anion | Expected mass (*m/z*) | Obtained mass (*m/z*) | Expected mass (*m/z*) | Obtained mass (*m/z*) |
| [N_4444_][ANS] | C_16_H_36_N^+^ | C_16_H_12_NO_3_S^-^ | 242.2842 | 242.2848 | 298.0543 | 298.0548 |
| [N_6666_][ANS] | C_24_H_52_N^+^ |  | 354.4094 | 354.4109 |  | 298.0547 |
| [P_4444_][ANS] | C_16_H_36_P^+^ |  | 259.2549 | 259.2562 |  | 298.0547 |

**Table S1.** ESI-MS analysis of synthesized compounds.

| **GUMBOS** | **T_50%_ (ºC)** | **Melting point (ºC)^a^** |
| --- | --- | --- |
| [N_4444_][ANS] | 308 | 108 |
| [P_4444_][ANS] | 422 | 95 |

## **Table S2.** Half weight loss temperature (T_50%_) and melting point of ANS-based GUMBOS.

^a^ For [NH_4_][ANS], m.p. = 237 ºC (TCI America).

# **References**

1. NMRShiftDB2 database. “Spectral Data”. https://nmrshiftdb.nmr.uni-koeln.de/portal/

js_pane/P-Results/nmrshiftdbaction/showDetailsFromHome/molNumber/20208017 [accessed Feb 14 2024].

2. PubChem. “8-Anilino-1-naphthalenesulfonic Acid”. https://pubchem.ncbi.nlm.nih.

gov/compound/8-Anilino-1-naphthalenesulfonic-acid [accessed Feb 14 2024].
